# Myopia in Children: Epidemiology, Genetics, and Emerging Therapies for Treatment and Prevention

**DOI:** 10.3390/children11121446

**Published:** 2024-11-27

**Authors:** Pier Luigi Surico, Uday Pratap Singh Parmar, Rohan Bir Singh, Yeganeh Farsi, Mutali Musa, Antonino Maniaci, Salvatore Lavalle, Fabiana D’Esposito, Caterina Gagliano, Marco Zeppieri

**Affiliations:** 1 Schepens Eye Research Institute of Mass Eye and Ear, Harvard Medical School, Boston, MA 02114, USA or psurico@meei.harvard.edu (P.L.S.);; 2 Department of Ophthalmology, Campus Bio-Medico University, 00128 Rome, Italy; 3 Department of Ophthalmology, Government Medical College and Hospital, Chandigarh 160030, India; 4 Department of Optometry, University of Benin, Benin City 300238, Nigeria; 5 Africa Eye Laser Centre, Km 7, Benin City 300105, Nigeria; 6 Department of Medicine and Surgery, University of Enna “Kore”, Piazza dell’Università, 94100 Enna, Italy; 7 Imperial College Ophthalmic Research Group (ICORG) Unit, Imperial College, 153-173 Marylebone Rd, London NW15QH, UK; 8 Department of Neurosciences, Reproductive Sciences and Dentistry, University of Naples Federico II, Via Pansini 5, 80131 Napoli, Italy; 9 Mediterranean Foundation “G.B. Morgagni”, 95125 Catania, Italy; 10 Department of Ophthalmology, University Hospital of Udine, 33100 Udine, Italy

**Keywords:** refractive errors, myopia, epidemiology, pediatrics, genetics, atropine, prevention

## Abstract

Refractive errors, particularly myopia, are among the most prevalent visual impairments globally, with rising incidence in children and adolescents. This review explores the epidemiology and risk factors associated with the development of refractive errors, focusing on the environmental and lifestyle factors contributing to the current surge in myopia. We provide an overview of key genetic factors and molecular pathways driving the pathogenesis of myopia and other refractive errors, emphasizing the complex interplay between genetic predisposition and environmental triggers. Understanding the underlying mechanisms is crucial for identifying new strategies for intervention. We discuss current approaches to slow myopia progression in pediatric populations, including pharmacological treatment regimens (low-dose atropine), optical interventions, and lifestyle modifications. In addition to established therapies, we highlight emerging innovations, including new pharmacological agents and advanced optical devices, and insights into potential future treatments. Cutting-edge research into gene therapy, molecular inhibitors, and neuroprotective strategies may yield novel therapeutic targets that address the root causes of refractive errors. This comprehensive review underscores the importance of early intervention and highlights promising avenues for future research, aiming to provide pediatricians with guidance to ultimately improve clinical outcomes in managing and preventing myopia progression in children and young adults.

## 1. Introduction

Myopia in children has emerged as a global public health concern, with its prevalence escalating at an alarming rate among various populations. This study seeks to establish a comprehensive framework that situates new findings within ophthalmic and public health literature, providing a practical resource for pediatric practitioners. This review aims to connect the epidemiological insights from past analyses with therapeutic applications in pediatrics. We seek to offer a resource that elucidates significant epidemiological trends and their ramifications for pediatric myopia therapy by analyzing the convergence of genetic, environmental, and lifestyle factors.

The complex roots of myopia, including genetic susceptibility and environmental as well as lifestyle factors, necessitate that doctors employ a comprehensive approach involving prevention, early detection, and effective intervention measures [1]. Pediatric care professionals are particularly positioned to undertake public health initiatives addressing the modifiable risk factors associated with the emerging evidence associating prolonged near work, less outdoor activity, and heightened digital screen usage with the beginning and progression of myopia [2]. Since early intervention in myopia significantly diminishes the likelihood of severe, vision-threatening complications in later life, pediatricians are crucial in informing families about preventive measures, ensuring compliance with treatment protocols, and coordinating prompt referrals to ophthalmologists.

Refractive errors, specifically myopia (nearsightedness) and hyperopia (farsightedness), are the result of irregularities in the axial length of the eye or its refractive components, such as the cornea and lens, which alter the focal point of light entering the eye [3]. In myopia, excessive axial elongation or an overly curved cornea causes light to focus anterior to the retina, leading to blurred distance vision [4]. In hyperopia, conversely, a shorter axial length or insufficient corneal curvature results in light focusing posterior to the retina, impairing vision [3]. These refractive errors are among the most common ocular conditions worldwide and, if left uncorrected, can lead to significant visual impairment and reduced quality of life. Early detection and correction of visual impairments in children are essential for preventing specific developmental delays such as difficulties in learning, social interaction, and motor skills. Addressing these issues during critical periods of visual maturation not only optimizes visual function but also supports overall cognitive and emotional development. Timely interventions can significantly reduce the risk of long-term consequences associated with uncorrected vision problems [5]. We aim to provide a comprehensive overview of the current mechanisms underlying myopia development in children, as well as to explore existing and emerging strategies for its prevention and treatment. We aim to inform clinicians and researchers about effective interventions and foster a deeper understanding of myopia management.

This study focuses on developing medicines and non-invasive public health measures in pediatric healthcare. We emphasize preventive methods that promote behavioral and environmental modifications, including enhanced outdoor exercise and screen time regulation, in conjunction with evaluating pharmaceutical and optical therapies that have been proven effective in managing myopia progression. Our study synthesizes findings on pharmaceutical therapies, such as low-dose atropine, with current data on optical and behavioral techniques, providing pediatricians with practical, evidence-based guidelines for routine patient counseling. Pediatricians frequently serve as the initial resource for families addressing myopia-related choices; therefore, they must possess knowledge of treatment effectiveness and adverse consequences, as well as strategies for successful communication and family education [6].

Our methodology emphasizes the necessity of harmonizing medical therapy with preventive efforts, as prevention is one of the most productive methods for tackling pediatric myopia on a population scale. This discovery has practical consequences for both individual and public health, seeking to diminish the incidence of myopia in future generations. We aim to empower physicians to proactively treat myopia by offering a resource that integrates evidence-based techniques with actionable information specifically designed for the pediatric healthcare environment. This not only improves their ability to advise families effectively but also promotes the long-term visual health of children as they develop.

## 2. Methods 

This narrative review was conducted through a comprehensive literature search using PubMed. We employed a range of keywords combined with Boolean operators to ensure a thorough examination of the relevant literature. The search terms included “refractive errors”, “myopia”, “epidemiology”, “pediatrics”, “genetics”, “atropine”, “prevention”, “gene”, “pediatric”, “children”, and “treatment”.

The search was designed to capture a wide array of studies and reviews addressing the various aspects of myopia, including its etiology, prevalence, and management strategies, particularly in pediatric populations. Relevant articles published in English were included based on their relevance and contribution to the current understanding of myopia. This approach allowed us to synthesize findings from diverse research areas, facilitating a comprehensive overview of the topic.

## 3. Epidemiology and Risk Factors

The prevalence of pediatric myopia has escalated worldwide in recent decades, establishing it as a critical public health issue, especially in urban and highly developed areas where lifestyle variables significantly influence its emergence and advancement [7]. This increase is not exclusive to industrialized regions; emerging nations are also witnessing a rise in childhood myopia, influenced by comparable behavioral and environmental risk factors [8]. Lifestyle and environmental factors are important. The COVID-19 pandemic highlighted the significant impact of environmental and lifestyle habits on the likelihood of myopia [9,10]. Extended durations of indoor confinement, elevated screen exposure, and diminished outdoor engagement have been recognized as significant risk factors for myopia [11]. Research indicates that children’s participation in near-work activities—such as reading, utilizing digital devices, and studying—can markedly elevate the risk of myopia development, particularly when conducted for extended, continuous durations [12]. Conversely, research consistently indicates that increased outdoor time confers a protective effect against myopia due to enhanced light exposure that increases dopamine release in the retina, hence aiding in the regulation of eye growth and axial elongation. However, the exact underlying mechanisms have not been fully understood [13]. Pediatricians can utilize this knowledge to advise families on including breaks during screen usage and encouraging balanced exposure to natural light as preventive measures against myopia development.

This condition not only affects visual acuity but also serves as a substantial risk factor for various sight-threatening complications, including glaucoma, myopic macular degeneration, and retinal detachment. The increasing prevalence of myopia in pediatric populations highlights the need for early detection and intervention, as the associated risks can lead to severe long-term visual impairment and reduced quality of life [14]. The prevalence of myopia is increasing globally [15], though different ethnicities are not equally affected, and the prevalence and burden of myopia among children vary across the globe [16,17]. While East Asian countries, like China, report a high prevalence of the disease, ranging from 37.7% to more than 80.0% in pediatric patients [18,19,20], the burden of myopia among children in Africa is significantly lower at around 4.7% [21]. The prevalence of myopia in the United States and Australia also varies considerably, reported at 41.9% and 24.8%, respectively [22,23]. There are also heterogeneous reports from European countries; for example, the estimated prevalence of myopia in Swedish schoolchildren aged 8–16 years has been estimated around 10%, while a study from Portugal in adolescents ranging from 10 to 18 years shows nearly double that, at over 21% [24,25].

There is an ongoing debate regarding the role of socioeconomic status and gender differences in childhood myopia. While children from higher socioeconomic backgrounds may have more access to digital devices and thus experience more screen time and near-work activities, those from lower socioeconomic backgrounds may lack adequate health evaluations, potentially leading to more severe cases of myopia being undetected [26]. There is also controversial evidence about gender differences. Although many studies report a higher prevalence of myopia in girls, likely due to reduced outdoor activities and increased near-work activities [18,20,27], some studies find no significant difference between boys and girls [25,28].

Genetic predisposition needs to be considered. Genetics greatly influence the development of myopia since the offspring of myopic parents have a markedly elevated risk of acquiring the condition. The heredity of myopia is extensively documented, with research demonstrating that children with two myopic parents have a 60% likelihood of developing myopia, in contrast to an estimated 20% risk in children without a familial predisposition [29,30]. Identifying familial risk factors enables pediatricians to conduct early screenings and implement preventative strategies, particularly for high-risk populations [31]. Research is actively investigating genetic markers linked to myopia, perhaps facilitating early identification of at-risk individuals through future screening systems. However, practical implementation in clinical pediatrics is still nascent.

Public health organizations, such as the World Health Organization (WHO) and ophthalmological associations, have acknowledged the diverse risk factors for myopia and have proposed preventive measures to mitigate high-risk behaviors. The WHO guidelines and research in East Asia recommend a minimum of 2 h of outdoor activity daily as a preventive strategy, indicating that children participating in such activities are 20–30% less likely to acquire myopia [32]. Additionally, numerous countries have initiated programs to inform parents and children about the effects of screen time and near-work activities, advocating for restrictions on digital device usage, particularly among young children [1,33]. Pediatricians are essential in public health campaigns, serving as educators and advocates for behavioral changes during routine consultations and community outreach efforts.

## 4. Pathogenetic Mechanisms of Refractive Errors

Refractive errors, including myopia and hyperopia, occur when the eye cannot correctly focus images onto the retina, leading to blurred vision. These conditions typically result from an imbalance between the eye’s optical components—mainly the lens and cornea—and the axial length of the eye. Myopia, also known as nearsightedness, and hyperopia, often known as farsightedness, are among the most prevalent refractive defects, along with astigmatism [34,35,36]. The pathogenesis of these conditions lies in a combination of genetic, epigenetic, and environmental factors [37]. 

Refractive errors are characterized by intricate genetic and molecular mechanisms [4,38]. Progress in genetic studies and molecular research has yielded vital insights into the mechanisms that underlie these disorders. Gaining a comprehensive understanding of these characteristics is of utmost importance to design successful interventions and to effectively manage the worldwide increasing impact of refractive errors.

### 4.1. Genetic Factors Contributing to Myopia

Myopia is a common disease in which distant things appear blurry because the images are focused in front of the retina. The degree of visual impairment determines whether this refractive error is classified as mild, moderate, or high myopia. The hereditary influence on myopia is extensively documented, with a greater occurrence reported in persons who have a familial background of the disease [39,40]. Studies on twins, especially those involving monozygotic twins, have provided additional evidence for the significant role of genetics in the development of myopia [41,42].

Genetic investigations have discovered many loci (*MYP1-MYP28*) linked to myopia, mostly through the inheritance pattern of autosomal dominance [43,44]. Nevertheless, these discoveries frequently lack reliable duplication, underscoring the intricacy of myopia genetics. Research on candidate genes has investigated the genes associated with the growth of the eye and the composition of the extracellular matrix. The *PAX6* gene, which plays a vital role in the development of the eyes, has been associated with both high and extreme myopia, highlighting its significance in the development of refractive errors [45].

Genome-wide association studies (GWASs) have enhanced our comprehension of the genetic makeup of myopia, revealing a considerable number of genomic polymorphic loci linked to the condition in various populations [46,47]. The CREAM consortium and 23andMe studies have played a crucial role in finding genes associated with neurotransmission, ion channel function, retinoic acid metabolism, and extracellular matrix remodeling. Some of the noteworthy genes are *GRIA4*, *KCNQ5*, *RDH5*, *LAMA2*, and *BMP2*, among others [48]. Although ongoing research is constantly providing valuable information, the precise mechanisms via which these genes contribute to the development of myopia have yet to be completely understood.

Recent progress in genetic research has discovered more related loci and potential genes that are linked to myopia, enhancing our comprehension of this intricate characteristic. In addition to the initial discovery of 28 loci, further genome-wide association studies (GWASs) have uncovered more than 160 genetic loci that are linked to refractive error. These loci include *PAX6*, *RBFOX1*, and *KCNMA1. RBFOX1*, for instance, has a role in the development of the nervous system and has been associated with high myopia in multiple research investigations. Furthermore, the *KCNMA1* gene, responsible for producing a large calcium-activated potassium channel, has been linked to the regulation of ocular growth [49].

Animal research has additionally yielded valuable information regarding the genetic pathways that underlie myopia. For example, experiments using mouse models have shown that mutations in the *Zfhx1b* gene cause alterations in the composition of collagen in the sclera, which in turn promote the development of myopic shifts. Furthermore, studies using gene knockout animals have demonstrated that the lack of the *GJD2* gene, which produces connexin 36, a protein involved in the retinal signals’ transmission, leads to impaired retinal development and myopia [46,50]. These findings indicate that genes involved in the transmission of nerve signaling and the restructuring of the extracellular matrix play a vital role in the development of myopia, which further emphasizes the complex character of this disorder.

Recent studies have employed advanced genetic and genomic techniques to deepen our understanding of myopia. Whole-genome and whole-exome sequencing has identified various genetic loci associated with myopia, highlighting the heritable nature of the condition [51]. Gene–environment interactions have been explored to understand how factors such as near work and outdoor activity influence genetic predispositions [52].

Mendelian randomization studies have provided insights into causal relationships between environmental factors and myopia risk, suggesting that increased time spent outdoors may have a protective effect [52,53]. Additionally, epigenetic research is beginning to reveal how environmental factors (i.e., education) can lead to changes in gene expression that may influence myopia progression [54,55]. Together, these studies underscore the multifactorial nature of myopia and suggest potential targets for prevention and treatment strategies.

Recent candidate gene studies in children have identified several genetic variants associated with myopia. For instance, variations in genes such as *MYOC* and *PAX6* have been linked to increased myopia risk [45,56,57]. These studies often focus on specific populations to uncover genetic predispositions that may interact with environmental factors. Additionally, research has examined the role of genes involved in eye development and refractive error, enhancing our understanding of myopia’s genetic underpinnings. These findings are crucial for identifying at-risk children and developing targeted interventions.

### 4.2. Molecular Mechanisms of Myopia Development

#### 4.2.1. Neurotransmitters and Growth Factors

The evolution of myopia is governed by intricate biochemical pathways that involve neurotransmitters and growth hormones. Dopamine, an essential neurotransmitter in the retina, controls the growth of the eye, and a decrease in its levels is linked to an increase in eye elongation and the development of myopia. The impact of light exposure on the production of dopamine indicates a connection between environmental factors and the advancement of myopia via the pathways of retinal dopamine [58].

Growth factors, such as transforming growth factor-beta (TGF-β) and insulin-like growth factor (IGF-1), are also important in the development of myopia. TGF-β influences the restructuring of the extracellular matrix in the sclera, which leads to the thinning of the sclera and the elongation of the eye’s axial length, both of which are characteristic features of myopia. Elevated concentrations of TGF-β have been detected in eyes with myopia, indicating its role in the development of the condition [59].

#### 4.2.2. Hypoxia and Signaling Pathways

Recent research indicates that the development of myopia may be influenced by hypoxia in the sclera. The activation of hypoxia-inducible factor 1α (HIF1α) stimulates the transformation of myofibroblasts, resulting in a reduction in collagen levels and a more pliable sclera. This, in turn, allows the elongation of the eye’s axis. This phenomenon can be worsened by decreased blood flow in the choroid, which further adds to the lack of oxygen in the sclera [60].

Furthermore, it has been suggested that signaling pathways, such as Wnt/β-catenin, have a role in controlling the growth of the eye in response to visual stimuli. These pathways affect the development of myopia by regulating different biological processes, such as cell proliferation and differentiation [60,61].

## 5. Approaches to Myopia Treatment and Prevention

This section offers pediatricians a succinct overview of myopia care alternatives, emphasizing effectiveness, availability, and possible adverse effects. Pediatricians should be well versed in both pharmacological and non-pharmacological treatment options due to the frequent concerns from parents regarding efficacy and safety. In this review we present a comparative analysis of the impact, side effects, and related risks of each intervention, facilitating pediatricians in delivering evidence-based recommendations.

### 5.1. Low-Concentration Atropine

#### 5.1.1. Mechanism of Action

The exact mechanism of action of atropine in myopia remains unclear. Initially, myopia was thought to result from excessive accommodation of the eye, leading to the use of atropine, which paralyzes the smooth ciliary muscles and induces cycloplegia [62]. This was believed to reduce accommodation and, in turn, slow myopia progression. However, recent research has shifted focus to non-accommodative mechanisms, exploring other molecular pathways through which atropine may act [63].

The current literature proposes multiple theories. Atropine sulfate, a muscarinic receptor antagonist, blocks the action of acetylcholine, thereby interfering with its role in regulating eye growth [64]. It has also been found to reduce epidermal growth factor receptor (EGFR) levels, which decreases cellular proliferation [65]. Since myopia development is linked to scleral thinning and remodeling, in vitro studies suggest that atropine reduces scleral fibroblast proliferation and increases the thickness of the scleral fibrous layer [66,67].

Another proposed mechanism involves increasing dopamine release, which helps to slow eye growth [68]. The choroid and retinal pigment epithelium (RPE) secrete growth factors, including transforming growth factor (TGF-β) and basic fibroblast growth factor (bFGF) [65]. Atropine is postulated to modulate eye growth by reducing the secretion of TGF-β through muscarinic receptor inhibition in RPE cells and lowering GABA transporter-1 (GAT-1) protein levels [69], which have been seen to play a role in eye growth and refractive development in animals [70].

#### 5.1.2. Dosing Regimens and Reported Side Effects

##### Historical Perspective 

The use of atropine to control the progression of myopia dates back several decades, with early research efforts providing a foundation for its role in treatment today. In the late 20th century, a landmark trial by Yen et al. in 1989 involved 96 children aged 6 to 14, where 1% atropine showed efficacy in slowing the progression of myopia. The study demonstrated a reduction in myopia progression to −0.22 diopters per year in the atropine group compared to −0.91 diopters in the placebo group. Axial length (AL) data were not available so the effect on axial elongation was not noted [71]. However, the widespread adoption of atropine was limited by significant side effects such as photophobia (light sensitivity) and blurred near vision, which were observed in many patients. This was a major drawback as it impacted daily activities like reading and outdoor play for children [71].

Further studies in the 1990s and early 2000s reinforced these findings. The studies by Brodstein et al. (1984) and Kennedy et al. (2000) followed large groups of children for extended periods (four years on average), further validating the effectiveness of atropine in slowing myopia progression [72,73]. Brodstein and colleagues followed 250 children for up to 9 years and observed a significant decrease in myopia progression in the treatment group using 1% atropine, once a day [72]. Similarly, Kennedy and colleagues in their cohort of over 200 kids, whom they followed for up to 11 years, reported significantly reduced myopia progression in the treatment group (0.05 D compared to 0.36 D per year in the control group) [73]. Additionally, these studies provided evidence that the degree of myopia progression in children treated with atropine was significantly lower than in those not receiving treatment, establishing atropine as one of the most effective methods available at the time.

In 1999, Shih and colleagues [74], in their RCT, investigated whether lower doses of atropine also had a similar effect on slowing myopia progression. They followed 200 children aged 6 to 13 years, on 0.5%, 0.25%, and 0.1% atropine and 0.5% tropicamide (as the control group) for 2 years. They concluded that the progression of atropine was least pronounced in the treatment group on 0.5% atropine eye drops (−0.04 ± 0.63 D/year), followed by the atropine 0.25% group (−0.45 ± 0.55 D/year) and the atropine 0.1% eye drop group (−0.47 ± 0.91 D/year) [74].

The most important advancement during this era came with the ATOM1 (Atropine for the Treatment of Childhood Myopia) study conducted by Chua and colleagues in 2006. This study involved 400 children and assessed the effect of 1% atropine in reducing myopia progression. The study’s results were significant: children treated with atropine had an average myopia progression of −0.28 diopters, compared to −1.20 diopters in the placebo group. Additionally, axial elongation—a key indicator of myopia progression—was reduced to almost zero in the atropine group compared to an increase of 0.38 mm in the placebo group [64].

However, while these results were encouraging, the side effects associated with 1% atropine continued to pose challenges. Photophobia and difficulty with near vision were common complaints [73], and the study also noted a rebound effect when treatment was stopped, with children in the atropine group experiencing accelerated myopia progression during the washout period [75].

##### Shift to Low-Dose Atropine

The search for a solution to the side effects associated with high-dose atropine led researchers to explore the possibility of using lower concentrations. Low-dose atropine (0.01%) has become a highly endorsed pharmaceutical alternative owing to its efficacy in decelerating myopia progression with reduced adverse effects. Pediatricians must apprise families of potential side effects, including mild photophobia and impaired near vision, even at this reduced dose [64,76,77]. This marked a shift in focus toward balancing efficacy with a reduction in side effects. One of the most important studies during this period was the ATOM2 study, published in 2012 [78]. ATOM2 compared three different concentrations of atropine, 0.5%, 0.1%, and 0.01%, for over 2 years. The study revealed that even at the lowest concentration of 0.01%, atropine was effective in slowing myopia progression and axial length elongation, with the added benefit of a significant reduction in side effects [78].

In ATOM2, children treated with 0.01% atropine experienced an average myopia progression of −0.49 diopters, compared to −0.76 diopters in those treated with 0.1% atropine and −1.14 diopters in those treated with 0.5%. Importantly, the side effects that had been observed with higher concentrations, such as photophobia and issues with near vision, were minimal in the 0.01% group [78]. Moreover, the rebound effect was much less pronounced in the 0.01% group compared to higher concentrations. Overall, after two years of treatment and the third year of the washout period, the overall progression of myopia was least in the 0.01% concentration group (−0.72 ± 0.72 D) [78]. The main limitation of this study was the lack of a placebo group.

Nevertheless, the results of the ATOM2 study marked a significant turning point in myopia management, leading to the widespread adoption of 0.01% atropine eye drops as a standard treatment for slowing myopia progression. In 2015, the WHO reported 0.01% atropine to be the most common strategy for the management of myopia in Asian countries like Singapore [79].

Building on the findings of ATOM2, the Low-concentration Atropine for Myopia Progression (LAMP) study [80], conducted in Asia, provided further evidence supporting the use of low-dose atropine. The LAMP study was a double-blind, randomized trial involving 438 children aged 4 to 12. It tested concentrations of 0.05%, 0.025%, and 0.01% atropine over one year and demonstrated that 0.05% atropine had the most significant impact on slowing myopia progression, followed by 0.025% and 0.01%. The study showed that higher concentrations of low-dose atropine had better efficacy, with the 0.05% group showing a reduction in myopia progression of −0.27 D compared to −0.81 D in the placebo group [80]. The key advantage of lower concentrations was the minimal impact on accommodation and photophobia. In the LAMP study, the side effects commonly associated with higher doses of atropine were rarely observed, making these lower doses more tolerable for children.

##### Current Opinion

Today, low-dose atropine is widely regarded as one of the most effective treatments for controlling myopia progression in children, particularly when considering both efficacy and side effects. Numerous studies have contributed to the current understanding of low-dose atropine, further solidifying its role in myopia management.

One of the notable studies in recent years is the CHAMP (Childhood Atropine for Myopia Progression) study [77], an international, placebo-controlled trial that analyzed the effects of 0.01% atropine on children aged 3 to 17 in the U.S. and Europe. This study demonstrated that low-dose atropine was effective across a wide demographic range and led to a significant reduction in myopia progression over three years. It provided the most robust evidence to date that low-dose atropine (particularly 0.01%) can significantly slow myopia progression with minimal side effects, such as blurred vision or light sensitivity, and with fewer concerns about the rebound effect upon cessation [77].

Another study conducted in California by Clark et al. in 2015 found that 0.01% atropine reduced myopia progression to 0.1 D per year rather than by 0.6 D per year in untreated control eyes [76]. Similar results were observed in a Spanish study, where children treated with 0.01% atropine with a follow-up period of 5 years experienced an average myopia progression of just −0.14 D annually, compared to −0.65 D in the placebo group [81]. Several studies conducted on cohorts from India, China, Japan, and Europe have also demonstrated the effectiveness of 0.01% atropine in slowing myopia progression, producing similar outcomes [82,83,84,85,86]. These consistent findings across diverse populations further reinforce the use of this concentration as a reliable treatment option for preventing and managing myopia progression globally. A summary of some of these studies and their findings are presented in Table 1.

##### Adverse Effects

Overall, the research indicates that while higher concentrations of atropine provide more significant control over myopia progression, they are associated with more adverse effects [95]. Qianwen and colleagues, in their meta-analysis of 19 studies involving 3137 children, identified the most common side effects of atropine use as photophobia (in 25.1% of participants), decreased near visual acuity (7.5%), and allergic reactions (2.9%) [96]. They further reported there to be a significant increase in the incidence of these side effects as the concentration of atropine increased (photophobia was reported in 6.3% with low-dose atropine, 17.8% with moderate-dose atropine and 43.1% with high-dose atropine). M.Y. Yen and colleagues reported that 100% of children receiving 1% atropine experienced photophobia [71]. In their clinical trial, Y.F. Shih and colleagues observed that lower concentrations of atropine were associated with fewer side effects: 22% of subjects on 0.5% atropine experienced photophobia, 7% on 0.25%, and only 0.25% on 0.1% [71].

Another notable side effect was the rebound phenomenon, where myopia progression accelerates—either through increased axial length (AL) or spherical equivalent (SE)—following the discontinuation of treatment. This effect is particularly associated with higher concentrations of atropine, contributing to their decline in clinical practice. Chia and colleagues found that this rebound effect was more pronounced with higher concentrations: 68% of children on 0.5% atropine, 59% on 0.1%, and only 24% on 0.01% experienced a myopic progression greater than 0.5 D [75]. Elevated intraocular pressure, potentially leading to atropine-induced glaucoma, is another reported side effect, but the risk is estimated to be as low as 0.005% [97].

### 5.2. Orthokeratology (Ortho-K)

Pediatricians should inform families that orthokeratology and multifocal lenses can effectively manage myopia through non-pharmacological means. These methods necessitate diligent compliance and hygiene, especially in younger children. While these procedures are mostly safe, the potential for consequences, such as microbial keratitis, must be conveyed [98,99].

Orthokeratology involves wearing specially designed lenses overnight to reshape the cornea, flattening the central area while steepening the mid-peripheral region [100]. This process addresses the concept of relative peripheral refraction, which is the difference in refraction between the central and peripheral retina [101]. When there is an optical defocus, it indicates a mismatch between the retina and the image plane [102]. Research has shown that unaided myopic eyes typically exhibit relative peripheral hyperopic refraction [103,104]. Orthokeratology corrects this by shifting the peripheral defocus from hyperopic before treatment to myopic afterward [105]. Studies suggest that this change is mainly driven by the degree of central myopia correction rather than a combination of central and peripheral defocus [106]. Overnight use of Ortho-K is particularly beneficial for those who prefer not to wear glasses or contact lenses during the day or for activities like sports, where optical corrections may be inconvenient. Overall, orthokeratology is considered a safe approach for myopia correction up to 6.00 D, with modern lenses designed for better centration and made from highly oxygen-permeable materials that allow for safer overnight wear [107].

#### 5.2.1. Effectiveness in Controlling Myopia Progression

Orthokeratology has proven effective in controlling myopia progression, with several studies showing reduced axial length growth in treated children. A two-year study in Hong Kong involving 35 children aged 7–12 years found that the mean axial length increase in the Ortho-K group was 0.29 mm, significantly lower than the 0.54 mm in the control group, indicating a 46% reduction in myopia progression [108]. Similar studies in the USA [109] and Japan [110] reported reduced axial length growth of 0.32 mm and 0.39 mm in children using Ortho-K compared to control groups. A study in Spain with children aged 6–12 years showed a 0.47 mm increase in axial length in the Ortho-K group versus 0.69 mm in the control group, reflecting a myopia progression reduction of 36% [111].

The effectiveness of Ortho-K extends to children with corneal astigmatism, with toric orthokeratology lenses being recommended for those with over 1.50 D of astigmatism for better centration and unaided daytime vision [112,113]. The Toric Orthokeratology for Slowing Eyeball Elongation (TO-SEE) study, conducted in Hong Kong, demonstrated that the mean axial length increase over two years in children treated with Ortho-K was 0.30 mm, significantly lower than the 0.64 mm increase observed in the control group [113].

#### 5.2.2. Potential Side Effects and Complications

Like other contact lenses, Ortho-K can lead to mild complications such as corneal staining, conjunctival hyperemia, corneal erosions, and papillary conjunctivitis [98]. Microbial keratitis, a serious sight-threatening condition, is also associated with contact lens wear, including Ortho-K, particularly due to poor hygiene or improper care routines [114]. Another challenge of wearing orthokeratology (Ortho-K) lenses is the potential compromise in visual quality, including reduced contrast sensitivity, decreased modulation transfer function (MTF) values, and increased aberrations [115]. This is thought to result from elevated scatter caused by uneven corneal refraction within the optical zone [116]. Additionally, studies have also shown that children who discontinue Ortho-K treatment tend to resume the natural progression rate of myopia [117].

#### 5.2.3. Combination Therapy with Atropine

Wang Z and colleagues, in their meta-analysis, compared the efficacy and safety of atropine therapy combined with orthokeratology to atropine or Ortho-K alone. They found that while Ortho-K and atropine alone had similar effects on AL (weighted mean difference (WMD) = 0.00 mm, *p*-value = 0.031), the combination regime significantly lowered AL when compared to these groups (WMD = 0.12 mm *p* value = 0.001) [84]. Another meta-analysis reported similar findings wherein they found the Ortho-K and low-dose atropine group to significantly lower the AL (WMD = 0.12, *p* < 0.001) and SE (WMD = 0.15 D, *p*-value < 0.001) when compared to Ortho-K alone [118].

### 5.3. Other Interventions

Animal studies have demonstrated that bright light exposure can completely prevent myopia development in form-deprivation models [119]. Increased outdoor time, especially in nonmyopic children, significantly reduces the risk of myopia onset and progression, with protective effects linked to exposure duration and light intensity. A randomized controlled trial showed that test groups (−0.84 D; 0.55 mm) had less myopic shift and axial elongation compared to controls (−1.04 D; 0.65 mm) [120]. Additionally, a meta-analysis found that near-work exposure raises the odds of myopia by 26% in workers, 31% in children, and 21% in adults [121]. In 2018, the Chinese Ministry of Education released the Comprehensive Plan to Prevent Near-sightedness among Children and Adolescents (CPPNCT) and recommended increasing time outdoors and reducing near work as effective interventions to decrease the incidence and progression of myopia in the young population [122].

Other interventions for myopia control include gas-permeable contact lenses and multifocal spectacles. Katz and colleagues reported no difference in myopia progression (*p* = 0.64) or axial elongation (*p* = 0.38) between children wearing gas-permeable and soft contact lenses over two years [123]. However, Walline and colleagues observed that gas-permeable lens wearers had slightly less myopia progression (−1.56 D) compared to soft lens wearers (−2.19 D), though there was no significant difference in axial elongation between the two groups [124]. Multifocal spectacles, designed to reduce accommodative effort, showed only a minor reduction in myopia progression, with children wearing multifocal lenses progressing 0.20 D less than those wearing single-vision lenses (*p* = 0.004), but this small difference over three years was not considered clinically significant [125]. Soft bifocal contact lenses, which provide additional power in the peripheral part of the lens, have also been studied for myopia control. These lenses reduced myopia progression by 46%, like the 43% reduction observed with orthokeratology lenses [126,127].

### Multifocal Contact Lenses (MFCLs)

Multifocal contact lenses help control myopia by reducing the peripheral hyperopic defocus and delivering a 360-degree peripheral myopic defocus [128]. Desheng Song and colleagues, in their meta-analysis, found that multifocal soft contact lens use in myopes was associated with a significantly reduced axial length elongation (mean difference (MD) = −0.08, *p*-value < 0.001) along with significantly reduced refraction progression (MD = 0.20 D, *p*-value < 0.001) when compared to the control group [129]. Another RCT in Taiwan reported a significant reduction in SE (WMD = 0.12 ± 0.34 D, *p*-value = 0.012) and AL (WMD 0.08 ± 0.010 mm, *p*-value < 0.001) growth in myopic school children wearing MFCLs when compared to the placebo group [130].

### 5.4. Advances in Myopia Control and Emerging Therapies

Liu and colleagues conducted a study on low-level red-light (RLRL) therapy for premyopic Chinese children, observing significant increases in choroidal thickness (CT) and choroidal vessel volume (CVV) in the parafoveal and perifoveal regions over 12 months. The therapy resulted in a slight hyperopic shift and axial length (AL) shortening early on, accompanied by steady foveal CT thickening [131]. Mohamed Ashraf Youssef and colleagues in their meta-analysis reported a statistically significant difference in AL and SE with RLRL in myopic individuals during 3-, 6- and 12-month follow-up intervals [132]. Another RCT observed that daily use of 650 nm low-level red light can slow myopia progression by significantly reducing the change in AL (MD = 0.19 mm CI, 0.16–0.22 mm, *p*-value < 0.001) and SER (MD = −0.41 D CI, −0.48 to −0.34, *p*-value < 0.001) [133].

Animal model studies have also reported that short-wavelength (violet) light inhibits myopia in mice by slowing refractive eye growth and producing hyperopic responses [134]. Another study by Hidemasa Torii and colleagues reported that violet-light-transmitting phakic intraocular lenses suppressed myopia progression and axial length elongation and can be considered as an intervention for human adults with high myopia [135].

Form-deprivation myopia (FDM) studies in guinea pigs showed reduced choroidal vasculature and lower VEGFA expression, which is crucial for choroidal blood flow. In these models, Conbercept treatment (a recombinant fusion protein inhibiting the VEGF pathway) increased myopia progression and axial length, suggesting that decreased VEGFA accelerates myopia.

Jiang and colleagues found that PAX6 gene methylation may affect myopia onset and progression in Chinese adolescents [136]. Additionally, EFEMP1, a regulator of the extracellular matrix, influences FDM through the PI3K/AKT/MMP2 axis, indicating that modifying EFEMP1 levels might help in preventing and treating myopia by affecting scleral remodeling [137].

In lens-based interventions, the MyoVision spectacle lenses, designed to reduce relative peripheral hyperopic defocus, successfully slowed myopia progression by 30% in children with a parental history of myopia, though it did not affect axial length elongation [138]. However, in another study in the Japanese cohort, the mean changes in SER and AL were not significantly different compared to the control group, so further studies are needed to verify the therapeutic potential of these spectacle lenses [139].

The DIMS (Defocus Incorporated Multiple Segment) spectacle lenses, which create myopic defocus across the retina, retarded myopia progression by 52% and axial length elongation by 62% over two years in Chinese children [126,140]. Diffusion Optics Technology (DOT) spectacle lenses use contrast modulation to control myopia by scattering light to reduce retinal contrast, potentially slowing eye growth [141]. The Control of Myopia Using Peripheral Diffusion Lenses Efficacy and Safety Study (CYPRESS), evaluating DOT spectacle lenses for myopia control, reported significant differences in SER (MD = −0.40 D, *p*-value < 0.001) and AL (MD = 0.15 mm, *p*-value < 0.001) between the spectacle lens and control group, verifying the efficacy of this modality [141].

Delaying myopia onset by one year has been shown to reduce the overall magnitude of myopia by 0.75–1.00 D, which is similar to the effects of 2–3 years of myopia management strategies, and pediatricians could play an important role in this [142]. An RCT conducted by Jinhua Bao and colleagues evaluated the efficacy of lenses with higher lenslet asphericity. They reported a difference of 0.34 mm in AL progression and 0.66 D in SE when comparing these lenses to single-vision spectacle lenses, resulting in better myopia control [143]. Paul Chamberlain and colleagues studied the effect of dual-focus contact lenses on reducing myopia progression. They found that eye growth was slowed by 71% in the treatment group compared to the control group [144]. Another study by Nicola S Anstice and colleagues also validated the efficacy of dual-focus contact lenses as they reported a significant decrease in AL (MD = 0.22 ± 0.10 mm, *p*-value < 0.001) and SE (MD = −0.44 ± 0.33 D, *p*-value < 0.001) when comparing these lenses to single-vision distance lenses [127].

## 6. Public Health Interventions and Behavioral Modifications for Myopia Prevention

Pediatricians play an active role in myopia prevention. Pediatricians are particularly equipped to incorporate these epidemiological findings into patient care, highlighting the significance of lifestyle adjustments within a comprehensive preventative strategy for myopia. They can collaborate with families to create daily routines that emphasize outdoor activities and regulate screen usage in developmentally suitable manners [145]. This preventative guidance corresponds with the pediatric healthcare model, which emphasizes early intervention and lifestyle counseling to enhance long-term health outcomes. For example, advocating for outdoor activities instead of screen-based entertainment can markedly diminish the risks of myopia progression while promoting general physical well-being [146].

Research reveals that each additional hour of daily outdoor activity correlates with a nearly 20% reduction in myopia incidence; therefore, physicians can provide families with structured recommendations for balancing indoor and outdoor activities [13]. Children should engage in a 10 min outdoor respite following every 50 min of near-work or screen activity to mitigate eye strain and promote healthier visual practices [12]. Organized outdoor activities, such as structured sports or family walks, can effectively fulfill these guidelines in a manner that is sustainable and compatible with family living.

Initiatives aimed at enhancing parental knowledge regarding the effects of screen usage are demonstrating encouraging outcomes in mitigating myopia risk. Pediatricians ought to advise parents on age-appropriate restrictions for screen time—typically no more than one hour daily for children under five, restricted to instructional content—and promote co-viewing to enhance the quality of screen interactions [33]. Through the discussion of these principles during regular appointments, physicians can provide parents with effective techniques to implement these adjustments and mitigate their children’s long-term risk of myopia.

Involving communities and schools in the adoption of these preventive measures is essential, especially in metropolitan regions where children’s access to outside environments may be restricted. Pediatricians can advocate for or establish school-based initiatives that encourage outdoor recess and minimize near-work requirements [32]. Research demonstrates that schools implementing compulsory outdoor breaks and organized outside activities have shown significant reductions in myopia development among pupils, highlighting the necessity of collective community initiatives.

The intricate interaction of genetic, environmental, and behavioral risk factors in pediatric myopia highlights the necessity for a proactive, community-focused preventative strategy. Pediatricians can considerably reduce the prevalence and progression of myopia in children by promoting balanced outdoor exposure, restricting screen usage, and teaching families about the hazards linked to excessive near work. This strategy assists individual patients while also aligning with overarching public health objectives to mitigate the global myopia epidemic [146].

Preventing myopia in children is greatly enhanced by public health and behavioral strategies that target lifestyle variables associated with the beginning of myopia. Numerous studies, including those examined in the National Academies’ assessment, indicate a strong correlation between heightened outdoor activities and reduced myopia prevalence. Pediatricians are ideally situated to promote behavioral changes, including advocating for outdoor play, minimizing screen usage, and regulating near-work activities [147,148]. Interventions encouraging a minimum of 2 h of outdoor activity daily have demonstrated a significant decrease in the onset and progression of myopia, offering a non-pharmacological strategy that coincides with the objectives of parents and pediatricians [147].

Moreover, several pharmaceutical therapies, such as low-dose atropine, effectively manage myopia progression; nevertheless, they may include potential adverse effects that clinicians ought to convey to parents. In areas with restricted access to ophthalmology services, primary care clinicians may assume a pivotal role in promoting these behavioral therapies and enhancing clinical care for vulnerable people. Pediatricians can partner with public health organizations to inform families and communities about these preventive measures, potentially decreasing the incidence of myopia and related consequences in future generations.

## 7. Limitations

This narrative review has several limitations. Firstly, our literature search was confined to PubMed and may not encompass all relevant studies published in other databases or in non-English languages. Additionally, the use of Boolean operators and specific keywords may have led to the exclusion of pertinent research that employed different terminologies or focused on related but distinct topics. Furthermore, as a narrative review, our synthesis of the literature is inherently subjective and may be influenced by the selection of studies. Lastly, the rapid advancements in myopia research mean that some recent findings may not be included, potentially limiting the comprehensiveness of our review.

## 8. Conclusions

In conclusion, although pharmaceutical therapies such as low-dose atropine and optical approaches show promise, public health efforts are pivotal in reducing myopia prevalence on a broader scale. Pediatricians are urged to integrate these approaches with proactive behavioral counseling, situating myopia prevention within a comprehensive, family-oriented framework. Pediatricians can effectively assist families in controlling and preventing myopia in children by integrating evidence-based therapies with practical, lifestyle-oriented measures.

## Figures and Tables

**Table 1:** Different dosing regimens of atropine and their efficacy in controlling the progression of myopia.

Study	Follow-up	Age	Case (Atropine Arm)/Control	N	Myopic Progression	Difference in Axial Length (AL)	Difference in Refraction (SE)	*p* Value	Ref
M Y Yen et al. (Taiwan; 1989)	12 months	6–14 years	1%	32	43.8%	-	−0.22 ± 0.54 D	<0.05	[71]
			Control	32	93.8%	-	−0.91 ± 0.58 D		
Y F Shih et al. (Taiwan, 1999)	24 months	6–13 years	0.5%	41	39%	-	−0.04 ± 0.63 D	<0.01	[74]
			0.25%	47	51%	-	−0.45 ± 0.55 D		
			0.1%	49	58%	-	−0.47 ± 0.91 D		
			Control	49	92%	-	−1.06 ± 0.61 D		
R H Kennedy et al. (USA; 2000)	3.5 years (Median)	6–15 years	Atropine	214	-	-	−0.05 D/year	<0.01	[73]
			Control	194	-	-	−0.36 D/year		
Y F Shih et al. (Taiwan, 2001)	18 months	6–13 years	0.5%	76	-	0.22 (0.03) mm/year	−0.41 (0.07) D/year	<0.0001	[87]
			Multifocal Lens	75	-	0.49 (0.03) mm/year	−1.19 (0.07) D/year		
			Single-vision spectacles	76	-	0.59 (0.04) mm/year	−1.40 (0.09) D/year		
W H Chua et al. (Singapore; 2006) (ATOM1)	24 months	6–12 years	1% Atropine	200	-	0.02 (0.35) mm/year	−0.28 (0.92) D/year	<0.0001	[64]
			Control	200	-	0.38 (0.38) mm/year	−1.20 (0.69) D/year		
Chih-Kai Liang et al. (Taiwan; 2008)	8.28 (2.48) months	6–15 years	0.5% Atropine	23	-	-	−0.15 (0.15) D/year	-	[88]
			0.25% Atropine	22	-	-	−0.38 (0.32) D/year	<0.01	
			0.25% + acupoints	26	-	-	−0.21 (0.23) D/year		
W H Chua et al. (Taiwan; 2012) (ATOM2)	24 months	6–12 years	0.5%	161	-	0.27 (0.25) mm/2 years	−0.30 (0.60) D/2 years	<0.05	[75]
			0.1%	155	-	0.28 (0.27) mm/2 years	−0.38 (0.60) D/2 years		
			0.01%	84	-	0.41 (0.32) mm/2 years	−0.49 (0.63) D/2 years		
Audrey Chia et al. (Singapore; 2012)	24 months	6–12 years	0.5%	139	37%	0.14 ± 0.13 mm	−0.15 ± 0.30 D	0.02 (SE between 0.01% and 0.5%) 0.01 (AL between all 3 groups)	[75]
			0.1%	141	42%	0.14 ± 0.14 mm	−0.19 ± 0.30 D		
			0.01%	75	50%	0.21 ± 0.16 mm	−0.25 ± 0.32 D		
Shu Yi et al. (China; 2015)	12 months	7–12 years	1%	68	-	0.03 ± 0.07 mm	−0.32 ± 0.22 D	<0.0001 (SE) <0.0001 (AL)	[89]
			Control	64	-	0.32 ± 0.15 mm	−0.85 ± 0.31 D		
Wang et al. (China; 2017)	12 months	5–10 years	0.5%	63	-	−1.01 mm/year	−0.8 D/year	<0.01 (AL)	[84]
			Control	63	-	0.5 mm/year	−0.2 D/year		
Diaz-Llopis M and Pinazo-Duran MD (Spain; 2018)	60 months (5 years)	9–12 years	0.01%	100	2%	-	−0.14 ± 0.35 D	<0.05	[81]
			Control	100	21%	-	−0.65 ± 0.54 D		
Jason C Yam et al. (Hong Kong; 2019) (LAMP)	12 months	4–12 years	0.05%	102	30.4%	0.20 ± 0.25 mm	−0.27 ± 0.61 D	<0.001 (SE) <0.001 (AL)	[80]
			0.025%	91	48.4%	0.29 ± 0.20 mm	−0.46 ± 0.45 D		
			0.01%	97	56.2%	0.36 ± 0.29 mm	−0.59 ± 0.61 D		
			Control	93	75.8%	0.41 ± 0.22 mm	−0.81 ± 0.53 D		
Qin Zhu et al. (China; 2020)	24 months	6–12 years	1%	262	-	0.12 ± 0.10 mm	−0.21 ± 0.22 D	<0.05 (SE) <0.05 (AL)	[90]
			Control	308	-	0.39 ± 0.19 mm	−0.89 ± 0.23 D		
Shifei Wei et al. (China; 2020)	12 months	6–12 years	0.01%	76	51.3%	0.32 ± 0.19 mm	−0.49 ± 0.42 D	<0.001 (SE) 0.04 (AL)	[91]
			Control	83	69.9%	0.41 ± 0.19 mm	−0.76 ± 0.50 D		
Aicun Fu et al. (China; 2020)	12 months	6–14 years	0.02%	117	49.8%	0.30 ± 0.21 mm	−0.38 ± 0.35 D	<0.001 (SE) <0.001 (AL)	[85]
			0.01%	119	54.9%	0.37 ± 0.22 mm	−0.47 ± 0.45 D		
			Control	100	71.9%	0.46 ± 0.35 mm	−0.70 D ± 0.60 D		
Rohit Saxena et al. (India; 2021)	24 months	6–14 years	0.01%	732	-	-	−0.27 ± 0.14 D (12 months)	<0.001	[82]
							−0.24 ± 0.15 D (24 months)		
Zigin Wang et al. (China; 2023)	12 months	6–12 years	0.01%	200	-	−0.11 mm (−0.17–0.06)	0.24 D (0.11–0.37)	0.01 (AL) <0.01 (SE)	[92]
			Control	100					
Manuel Moriche-Carretero et al. (Europe; 2024)	60 months	6–12 years	0.01%	184	-	0.26 ± 0.28 mm	−0.63 ± 0.42 D	-	[86]
			Control	177	-	0.49 ± 0.34 mm	−0.92 ± 0.56 D		
Doville Simonaviciute et al. (2024)	12 months	6–12 years	0.03%	31	35.5%	0.19 (0.17) mm	−0.34 (0.44) D	0.024 (SE) 0.05 (AL)	[93]
			Control	41	61%	0.31 (0.20) mm	−0.60 (0.50) D		
Rohit Saxena et al. (India; 2024)	12 months	6–14 years	0.01%	47	13%	0.22 ± 0.20 mm	−0.16 ± 0.38 D	0.021 (SE) 0.19 (AL)	[94]
			Control	45	38%	0.28 ± 0.28 mm	−0.35 ± 0.40 D		
Osamu Hieda et al. (Japan; 2024)	24 months	6–12 years	0.01%	77	-	0.63 ± 0.04 mm	−1.26 ± 0.09 D	<0.001 (SE) <0.001 (AL)	[83]
			Control	81	-	0.77 ± 0.04 mm	−1.48 ± 0.09 D		

## Data Availability

Not applicable.
